# Enhanced Production of *β*-Nicotinamide Mononucleotide with Exogenous Nicotinamide Addition in *Saccharomyces boulardii*-YS01

**DOI:** 10.3390/foods12152897

**Published:** 2023-07-29

**Authors:** Meijie Song, Chunhua Yin, Qianqian Xu, Yang Liu, Haiyang Zhang, Xiaolu Liu, Hai Yan

**Affiliations:** School of Chemistry and Biological Engineering, University of Science and Technology Beijing, Beijing 100083, China; meijie_song@126.com (M.S.); chyin@ustb.edu.cn (C.Y.); qianqianxu@ustb.edu.cn (Q.X.); liuyang@ustb.edu.cn (Y.L.); zhanghy@ustb.edu.cn (H.Z.); xiaoluliu@ustb.edu.cn (X.L.)

**Keywords:** *β*-nicotinamide mononucleotide, nicotinamide, UPLC-ESI-QqQ-MS/MS, pathway, gene of *Saccharomyces boulardii*-YS01

## Abstract

*β*-Nicotinamide mononucleotide (NMN), as a key precursor of an essential coenzyme nicotinamide adenine dinucleotide (NAD^+^), is most recognized for its pathological treatment effects and anti-aging functions. Here, the biosynthesis of NMN from the inexpensive feedstock substrate nicotinamide (Nam) using previously isolated *Saccharomyces boulardii*-YS01 was investigated. Ultra-high performance liquid chromatography coupled to triple quadrupole tandem mass spectrometry (UPLC-ESI-QqQ-MS/MS) was established for the determination and targeted analysis of NMN, nicotinamide riboside (NR), nicotinic acid (NA), Nam, and NAD^+^ in YS01 cells. Satisfactory precision and accuracy values were achieved with recoveries above 70% for five analytes. A 5~100 times higher content of NMN in YS01 (0.24~103.40 mg/kg) than in some common foods (0.0~18.8 mg/kg) was found. Combined with genome sequencing and enzyme function annotation, target-acting enzymes, including nudC, ISN1, URH1, PNP, and SIR2, were identified, and the biosynthetic pathway of NMN via Nam was suggested. The initial addition of 3 g/L Nam in the culture medium effectively promoted the generation of NMN, which raised the content of NMN by 39%. This work supplements an alternative resource for NMN production and lays the theoretical foundation for the further construction of NMN transgenic synthesis hosts.

## 1. Introduction

*β*-Nicotinamide mononucleotide (NMN) is a kind of bioactive nucleotide compound which is naturally formed by the reaction between a phosphate group and a nucleoside-containing ribose and nicotinamide [1]. NMN exists in all living organisms and is generally located in the nucleus, mitochondria, and cytoplasm [2]. Primitively, NMN was mainly regarded as a source of cellular energy and a vital intermediate in nicotinamide adenine dinucleotide (NAD^+^) biosynthesis. Currently, there is mounting evidence that NAD^+^ deficiency is inextricably linked to a series of age-related pathophysiological disorders and diseases [3,4,5]. Numerous investigations have also demonstrated that boosting NAD^+^ through NMN supplementation is a convincing therapy for considerably ameliorating miscellaneous diseases, such as diabetes [6,7], cardiovascular diseases [8,9], acute kidney injury [10], Alzheimer’s disease [11], and sepsis-associated encephalopathy [12], among others. Above all, the oral administration of NMN can effectively compensate for poor absorption through the direct feeding of NAD^+^, where NMN crosses the cell membrane by active transport and is then converted into NAD^+^ in human cells [4]. Thus, as a promising nutritional supplement/nutraceutical, research on the production of NMN is crucial to enhancing human health.

Due to the limited efficiency and facile quality of industrial production methods, commercial products containing NMN are highly-priced. Existing chemical or enzymatic synthesis techniques often require costly substrates and particular chemicals, but microbial synthesis methods possess environmental friendliness and safety, which can greatly reduce costs [13,14]. Multiple attempts have been carried out to prepare NMN through biotechnological methods in various microorganisms. By and large, *Escherichia coli* is the most commonly used genetically engineered bacteria to generate NMN, and has made some progress in increasing yield and saving costs [13,14,15,16]. Studies on the synthesis of NMN in yeast are limited; some articles to date have primarily reported certain enzymes and particular paths involving NMN in *Saccharomyces cerevisiae* (*S. cerevisiae*) [1,4,17,18]. In *S. cerevisiae*, it has been concluded that NMN can be produced from nicotinamide riboside (NR) by nicotinamide riboside kinase 1 (Nrk1)-catalyzed phosphorylation and is then adenylylated to NAD^+^ by Nma1 and Nma2 (nicotinamide mononucleotide adenylyltransferase) [17]. Beyond that, the most recent report provides a stable and low-cost whole-cell biocatalyst named human nicotinamide riboside kinase 2 (Nrk2) which is functionally displayed on the cell surface of *S. cerevisiae* and can convert NR to NMN efficiently [19]. Plainly, yeast is also an excellent strain that is worthy of development and improvement.

As is widely known, *Saccharomyces boulardii* (*S. boulardii*) is the only yeast possessing probiotic with characteristics including antimicrobial activity, the production of antimicrobial peptides, immune modulation, and trophic effects, and can modulate the host microbiome and intestinal function. It has been incorporated into dietary supplements, which are available on the commercial market [20,21]. *S. boulardii* could be a potentially exploitable NMN production material. On one hand, despite *S. boulardii* being closely associated with *S. cerevisiae*, the distinctive traits of normal growth at human temperature, survival in low acidity environments, its inefficiency in using galactose as a carbon source, and inability to form ascospores give rise to a clear differentiation between *S. boulardii* and *S. cerevisiae* [22,23]. On the other hand, the way in which NMN levels might be regulated is not clearly understood in *S. boulardii*, even though previous studies pay the most attention to the metabolism of NAD^+^ in other carriers [24,25,26]. Hence, the biosynthesis and metabolic pathways of NMN in *S. boulardii* are yet to be further investigated.

Metabolomics is already widely recognized in scientific research as a robust, efficient, and sensitive analytical tool, whereas targeted analysis can provide absolute quantification of samples and allow for an association between the material and known well-characterized analytes to be observed [27,28]. Moreover, genetic information is inseparable from specific phenotypes, which can help in identifying and understanding the molecular basis of some physiological and biochemical behavior, and further supports genetic engineering approaches to transform organisms [29,30]. At present, a variety of methods have been applied to detect the NMN in different matrixes. Aside from the liquid chromatography method [31] and enzyme-coupled assay [32], liquid chromatography–tandem mass spectrometry is the more widely used method [24,25,26]. In this study, in order to accurately and quantitatively analyze and lock the NMN generation pathway, we developed and validated a reliable UPLC-ESI-QqQ-MS/MS method based on targeted metabolomics to simultaneously measure nicotinamide (Nam), NR, nicotinic acid (NA), NAD^+^, and NMN in *S. boulardii*-YS01 during different growth periods. Afterward, we identified the enzymes by gene annotation to obtain a proposed metabolic pathway. Additionally, our strategy to accelerate the formation of NMN in a simple and convenient manner was to add the appropriate concentrations of the aqueous Nam solution into the culture medium. The present study aimed to complement a NMN production resource, which provided a theoretical reference and ideas for the subsequent construction of strain gene engineering and the development of multi-functional compound health products.

## 2. Materials and Methods

### 2.1. Chemicals and Culture Medium

NMN (>95%), NR (≥95%), Nam (≥99.5%), and NA (≥99%) were purchased from Shanghai yuanye Bio-Technology Co., Ltd. (Shanghai, China); NAD^+^ (>98%) was purchased from Shanghai Macklin Biochemical Co., Ltd. (Shanghai, China). Ultra-pure water was prepared using a Milli-Q water system (Milli-pore–Waters, Milford, MA, USA). HPLC-grade methanol was obtained from Fisher Chemicals (Fairlawn, NJ, USA). Formic acid (MS-grade) and ammonium formate were obtained from Thermo Fisher Scientific (Shanghai, China).

The complex medium of *S. boulardii*-YS01 contained (g/L) glucose (10), saccharose (20), peptone (10), yeast extract (10), malt powder (1), Na_2_CO_3_ (0.3), KH_2_PO_4_ (1.5), MgSO_4_ (1), ammonium citrate tribasic (5), ammonium ferric citrate (0.02), glycerin (10), and multivitamin B tablet (0.1 piece/L). All of them were dissolved in deionized water and then filled to their final volume. The corresponding solid medium was prepared by adding 2% AGAR powder to the liquid medium. The media and vessels (e.g., Erlenmeyer flask and centrifuge tubes) were sterilized in an autoclave at 115 °C for 30 min.

### 2.2. Sample Preparation

*S. boulardii*-YS01 (Figure S1) was previously isolated and preserved in our laboratory. The sterilized medium was cooled down to room temperature, then inoculated with YS01 cells from the logarithmic growth phase (approximately 3~5 × 10^7^ CFU/mL tested by plate counting method, the inoculation amount was 1%) and incubated in the shaker at 32 °C and 200 rpm. In addition, by adding 1, 3, 5, and 10 g/L of Nam to investigate the optimum dosage that promoted the production of NMN in YS01, the Nam aqueous solution needed to be filter-sterilized and irradiated with ultraviolet light. For the detection of the NMN metabolic pathway, samples with or without the addition of Nam from different growth time points (0 h, 12 h, 24 h, 36 h, 48 h, 60 h, and 72 h) were selected for the test. The YS01 yield was measured and quantified at the growth stationary phase.

The samples for UPLC-ESI-QqQ-MS/MS analysis were prepared as follows: culture liquids of *S. boulardii*-YS01 cells (20 mL), obtained from different growth periods, were collected into a centrifuge tube (50 mL) and then centrifuged (10,000× *g*, 10 min). The supernatant was discarded after centrifugation, and the remaining cells were washed twice with deionized water, centrifuged (10,000× *g*, 10 min), extracted with 5 mL of ethanol and mixed vigorously for 5 min on a vortex mixer before sonicating them for 5 min. Next, 5 mL of ultra-pure water was added into the above centrifuge tube while mixing the cells vigorously for 30 min. After centrifugation (10,000× *g*, 10 min), 20 μL of the supernatant extracts were taken and diluted to 1 mL with ultra-pure water. Afterward, it was filtered through a 0.22 µm organic nylon micropore membrane (13 mm, 0.2 μm, Agilent, made in Beijing, China), then immediately subjected to UPLC-ESI-QqQ-MS/MS analysis.

### 2.3. UPLC-ESI-QqQ-MS/MS Analysis

UPLC-ESI-QqQ-MS/MS was performed using an Agilent 1290 UHPLC system that was connected to an Agilent 6495B QqQ MS (Santa Clara, CA, USA) tandem mass spectrometer, outfitted with an electrospray ionization source operating in the positive mode. An optimized method was established to analyze NMN, NR, NA, Nam, and NAD^+^ in the samples. The ion pairs of the above five compounds were determined, and the collision energy of each ion pair was optimized to obtain the highest response. The detection method employing ultra-high-performance liquid chromatography coupled with triple quadrupole mass spectrometry in a multiple reaction monitoring mode was then used to quantify the NMN, NR, NA, Nam, and NAD^+^ in *S. boulardii*-YS01 samples.

The detailed conditions were as follows: all compounds were separated over a reversed-phase chromatographic column (Waters X Select HSS T3, 2.1 × 100 mm, 2.5 μm) at 35 °C. The mobile phase consisted of 5 mM ammonium formate in water (containing 0.4% formic acid, volume fraction) (A) and methanol (B). The separation was performed using the following gradient: 0% B (0~3 min), 0~90% B (3~4 min), 90% B (4~7 min), 0% B (7.1 min), followed by equilibration at 0% B for 3 min. The flow rate at all times was 0.2 mL/min, and the injection volume was 2 μL. The drying gas was at 250 °C with a flow rate of 16 mL/min. The sheath gas temperature was set to 325 °C with a flow rate of 12 mL/min, and the nebulizer gas pressure was 35 psi. The capillary voltage was 3500 V with a nozzle voltage of 500 V. The Delta EMV (+) was 200, and the cell accelerator voltage was 3. Three sets of repeats were performed in UPLC-ESI-QqQ-MS/MS analysis. For quantification, under the condition of the multiple reaction monitoring (MRM) mode, an external standard method was applied, and the standard curve was built.

### 2.4. Method Validation

By eliminating the matrix effect and spiked recovery experiment to validate the methodology, the standard calibration curve was derived from six points (5 ng/mL, 10 ng/mL, 50 ng/mL, 100 ng/mL, 500 ng/mL, and 1000 ng/mL).

The supernatant extracts (Section 2.2) of *S. boulardii*-YS01 at the stationary phase were indicated as “dilution factor 10” due to there being no blank matrix sample. The dilution chart was as follows: the dilution factors ranged from 20 to 500 for YS01. The 20, 50, 100, 200, and 500-fold diluted extracts containing 20 μL of 10 mg/L mixed standards were obtained from a volume of 500, 200, 100, 50, and 20 μL of the dilution factor 10 samples into a final volume of 1000 μL Ultra-pure water. Corresponding standards in the solvent were prepared to calibrate the extracts’ concentration.

The matrix effect (ME) was described as the change in ionization efficiency in the presence of other compounds and was expressed as the response of the NMN, NR, NA, Nam, and NAD^+^ in the matrix compared to the signal in the solvent and were calculated by the following equation [33,34].

(1) $$\mathrm{ME}=\left(\frac{\mathrm{Peak}\;\mathrm{area}(\mathrm{spiked}\;\mathrm{extract})}{\mathrm{Peak}\;\mathrm{area}(\mathrm{solvent}\;\mathrm{standard})}-1\right)\times 100\%$$

A value of ME = 0% meant that no matrix effect occurred. Negative values represented the suppression of the analyte signal, and positive values stood for enhancements induced by the matrix. No matrix effect existed when the values were between −20% and 20%, the medium matrix effect was between −50% and −20% or 20%, and 50%, and a strong matrix effect was below −50% or above 50% [33].

Accuracy (recovery, %) and precision were estimated from the recovery experiments. The spiked samples were extracted under the above-mentioned extraction procedure in Section 2.2. About 1 g of *S. boulardii*-YS01 cells, after centrifuging and washing, was spiked with the mixed standard solution including NMN, NR, NA, Nam, and NAD^+^ at the level of 10, 50 and 100 mg/kg with three replications. The limit of detection (LOD) and limit of quantitation (LOQ) were defined as 3 and 10 times the signal/noise ratios (S/N), respectively. Each spiked level was determined 6 times in parallel.

### 2.5. Genome Scanning Sequencing and Analysis of Strain-YS01

The genome of the purified *S. boulardii* strain-YS01 was scanned using the Illumina high-throughput sequencing (Illumina Hiseq × 10 platform). DNA extraction, genome sequencing, and assembly were performed at Shanghai Majorbio Bio-Pharm Technology Co., Ltd. (Shanghai, China). The raw genome sequences of YS01 were deposited in the SRA of the NCBI database under the accession number OR077440. The nearest neighbor sequences were aligned using Clustal W, and a phylogenetic tree based on ITS rDNA sequence data were constructed by the neighbor-joining method with MEGA 11 (11.0.10) software.

Genomic DNA was extracted using the Omega Fungal DNA Kit D3390-02 according to the instructions. Purified genomic DNA was quantified by TBS-380 fluorometer (Turner BioSystems Inc., Sunnyvale, CA, USA). High-quality DNA (OD260/280 = 1.8~2.0, >1 μg) was used to conduct further research. For Illumina sequencing, at least 1μg of genomic DNA was used for each strain in the sequencing library construction. The 5′ prime ends were first end-repaired and phosphorylated, and the 3′ ends were A-tailed and ligated to sequencing adapters. The next step was to enrich adapter-ligated products using PCR. The prepared libraries were then used for paired-end Illumina sequencing (2 × 150 bp) on an Illumina HiSeq X Ten machine.

A statistic of quality information was applied for the quality trimming of original data, and an assembly of the clean reads was performed using SOAPdenovo2. The identification of predicted coding sequences (CDS) was performed using Maker2, tRNA-scan-SE was used for tRNA prediction, and Barrnap was used for rRNA prediction. The predicted CDSs were annotated from the Non-Redundant Protein Sequence Database, Swiss-Prot, Pfam, Clusters of Orthologous Groups (COG), Gene Ontology (GO), and Kyoto Encyclopedia of Genes and Genomes (KEGG) database using sequence alignment tools such as BLAST, Diamond, and HMMER. Briefly, each set of query proteins was aligned with the databases, and annotations of best-matched subjects (E-value < 10^−5^) were obtained for gene annotation.

### 2.6. Data Processing and Statistical Analysis

UPLC-ESI-QqQ-MS/MS raw data were acquired using Agilent MassHunter version B.10.00. MassHunter Quan Analysis 10.0 software was used for quantitative analysis. The statistical analysis was performed in MetaboAnalyst 5.0 and Origin 2021 software (OriginLab Inc., Northampton, MA, USA). All experiments were repeated in triplicate, and results were reported as means ± standard deviation. The statistical significance of data was analyzed by Duncan’s test, which was performed with the IBM SPSS Statistics 22 statistical package. A value of *p* < 0.05 was considered to be statistically significant.

## 3. Results and Discussion

### 3.1. Establishment and Validation of the Analytical Method

An accurate method for the determination of certain biologically active substances is core to monitoring the compound’s content and evaluating its function. This study established a quick and viable detection method so as to analyze the concentrations of NMN, NR, NA, Nam, and NAD^+^ in *S. boulardii*-YS01 samples. The method was validated in terms of linearity, LOD, LOQ, matrix effect accuracy, and precision.

The mass spectrometry conditions and gradient elution procedure were optimized and made suitable for quantitative mass spectrometry. The unique and stable quantitative and qualitative ion pairing suitable for use as MRM transitions are presented in Table 1. The chromatograms of MRM types, when extracted, showed a good separation of five analytes (Figure S2). In addition, many works in the literature have reported that the pure solvent dilution method can effectively improve the matrix effect during the determination of pesticides by LC-ESI-MS/MS [33,34]. Since the five compounds mentioned above are naturally occurring substances during the culture of *S. boulardii*-YS01, blank samples for the preparation of matrix matching and standard working solutions are not available. Based on this, a dilution-based method was conducted with the aim of diminishing the matrix effects. At a dilution factor of 500, the matrix effect of five compounds in the test samples was effectively controlled, basically fluctuating around −20~20% (Figure S3). Therefore, accurate quantitative results could be obtained by solvent standard curve correction.

The recoveries with relative standard deviations (RSDs) for each analyte were measured by spiking in YS01 samples at three different spiked levels (10, 50, and 100 mg/kg). The standards were dissolved with ultra-pure water and diluted to the appropriate concentrations, and the linearity of calibration curves constructed at six levels was acceptable with a regression coefficient (R^2^) higher than 0.998. The LOD obtained was between 0.001 µg/kg and 0.025 µg/kg and the LOQ obtained was between 0.003 µg/kg and 0.082 µg/kg, which showed a good detection limit and sensitivity compared with those of reported methods [25,26,32]. The mean recoveries ranged from 70 to 89%, with an RSD of 2.7~7.1%. The detailed parameters are shown in Table 2. These results indicated that the proposed method could achieve good quantification of NMN, NR, NA, Nam, and NAD^+^ in studied matrices.

### 3.2. UPLC-ESI-QqQ-MS/MS Quantitation of NMN, NR, NA, Nam and NAD^+^ in S. boulardii-YS01

Nam has been well known as a widely used and inexpensive raw material in the industry. NAD^+^ and its precursors, NR, NMN, Nam, and NA have been pointed to as potentially intriguing molecules in terms of geroprotectors and/or pharmacogenomics [18,35]. Elsewhere, the exogenous supplemented effects of Nam have been studied in both *S. cerevisiae* Replicative Lifespan (RLS) and Chronological lifespan (CLS) models in some reports, proving that Nam addition in the yeast growth medium can shorten RLS and promote CLS by the inhibition of Sir2 (NAD^+^-dependent protein deacetylases) activity [18]. Here, we attempted to add a certain concentration of Nam to the culture medium and observe its auxiliary role in NMN generation in *S. boulardii*-YS01.

**Figure 1:**
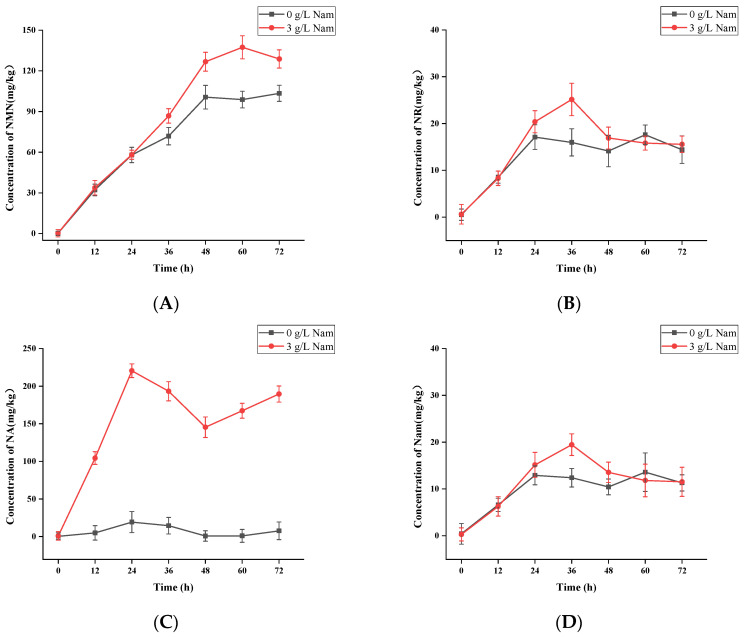

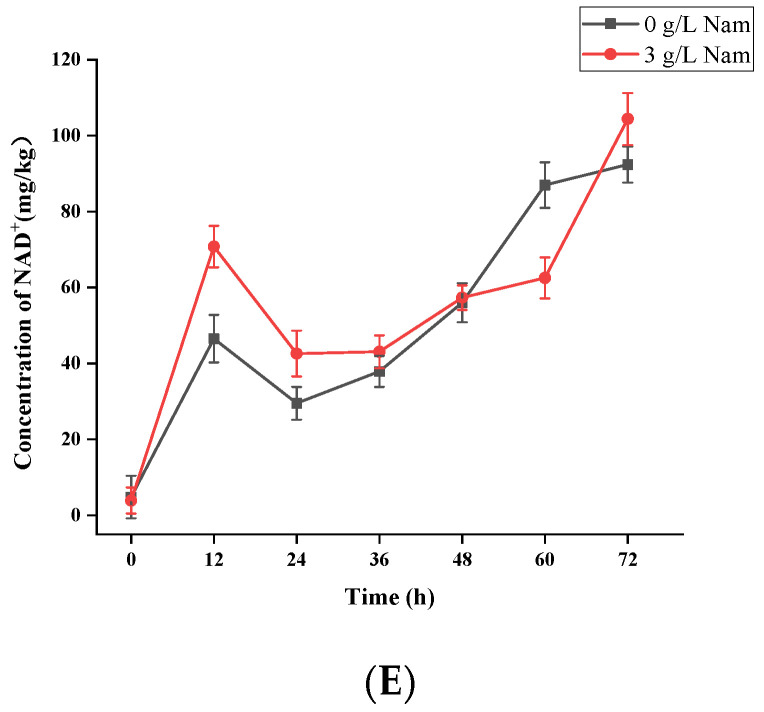
The changes in five analytes during different culture time in *S. boulardii*-YS01, (**A**) *β*-Nicotinamide mononucleotide (NMN), (**B**) Nicotinamide riboside (NR), (**C**) Nicotinic acid (NA), (**D**) Nicotinamide (Nam), (**E**) Nicotinamide adenine dinucleotide (NAD^+^).

**Figure 2:**
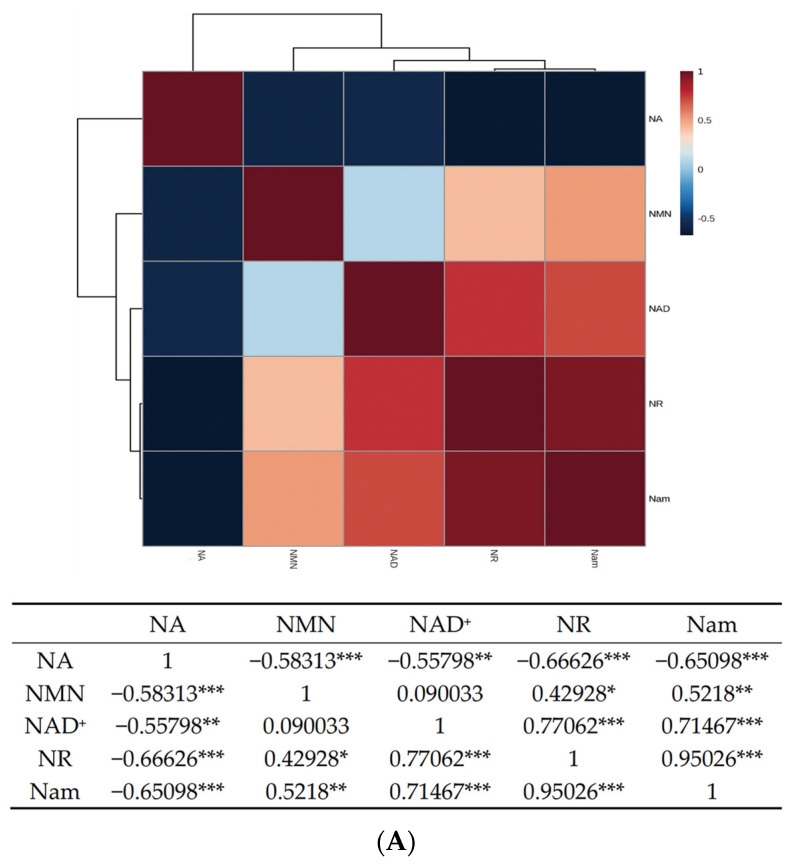

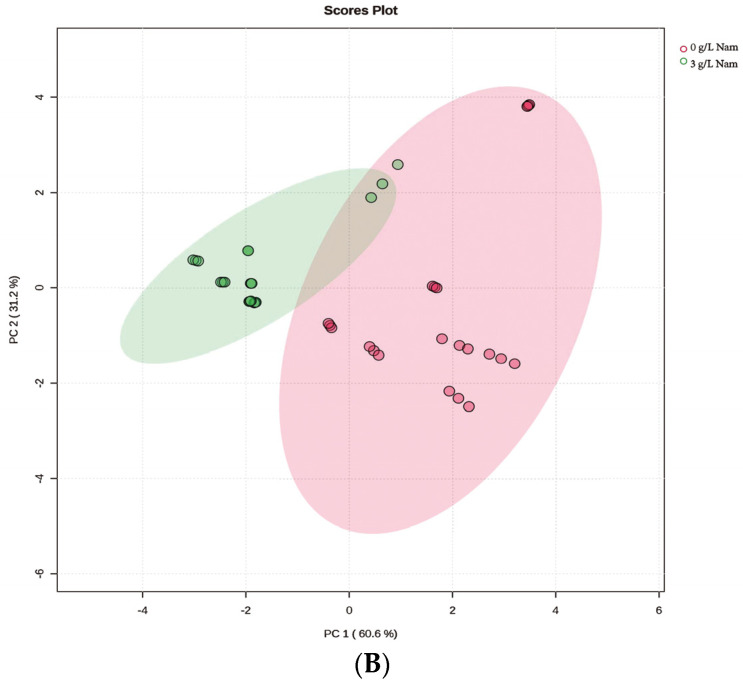
The relationship description between *β*-nicotinamide mononucleotide (NMN), nicotinamide riboside (NR), nicotinic acid (NA), nicotinamide (Nam) and nicotinamide adenine dinucleotide (NAD^+^) in *S. boulardii*-YS01. (**A**) Heatmap for Pearson’s coefficients among the five analytes. Asterisks mean significant differences (* means *p* ≤ 0.05, ** means *p* ≤ 0.01, *** means *p* ≤ 0.001), (**B**) PCA plot of *S. boulardii*-YS01 samples.

For the purpose of exploring the content and changes in the five analytes, samples without Nam addition were taken as the control group, and samples with 3 g/L of Nam added were taken as the experimental group. Two sets of samples collected from different culture time points were simultaneously determined and analyzed. Detailed information is illustrated in Figure 1 and Table S1. The principal component analysis (PCA) plot showed differences between the two groups of samples (Figure 2B). Through quantifying the NMN and for further comparison with the content of NMN surveyed and reported in some ordinary foods, including vegetables, fruits, and mushrooms, among others (0.0~18.8 mg/kg) [2,32], we found that the NMN (0.24~103.40 mg/kg) was at least 5~100-fold higher in *S. boulardii*-YS01. It is denoted that YS01 can be used as a functional product resource to supplement NMN. Furthermore, after adding 3 g/L Nam, the average level of NMN in YS01 after the addition of 3g/L Nam ranged from 0.26 to 137.36 mg/kg and was obviously raised by 20% to 40%. We thought that the addition of Nam might increase the production of NMN by promoting cell growth, whereas it turned out that Nam slightly inhibited the growth (OD_600_) of YS01 by measuring the optical density at 600 nm (Figure S4). The specific mechanism of this pends following explorations.

NA has been corroborated in the possession of good neuroprotective function, especially in neurodegenerative diseases [36]. The average level of NA was increased to 104.41~220.51 mg/kg during the culture process of YS01, hinting that the addition of Nam might be responsible for promoting the production of NA to 250 times the initial level (0.86 mg/kg). On the contrary, through the correlation analysis (Figure 2A), there was a significant negative correlation between Nam and NA. Not only that, but NA was also negatively correlated with NMN, NR, and NAD^+^; therefore, subsequent studies on the relevant mechanism are needed. Except for this, no apparent regular changes in NAD^+^ were found, and the levels of Nam and NR followed a similar trend in YS01. Overall, the quantification of NMN, NR, NA, Nam, and NAD^+^ highlights the potential of the stain-YS01 to act as biological factories for beneficial molecules to humans.

With reference to the correlation analysis (Figure 2A), there was a positive correlation among NMN, Nam, NAD^+^, and NR. Additionally, it was not hard to find that NMN was positively correlated with NAD^+^, NR, and Nam, particularly for the most significant positive correlation with Nam (*p* ≤ 0.01). Apart from this, NR was also significantly and positively correlated with Nam, and NAD^+^ was positively correlated with NMN, NR, and Nam. The above results imply that the complicated interaction mediated by Nam among the five analytes warrants further research. 

### 3.3. Overview of the S. boulardii Strain-YS01 Genome

**Figure 3:**
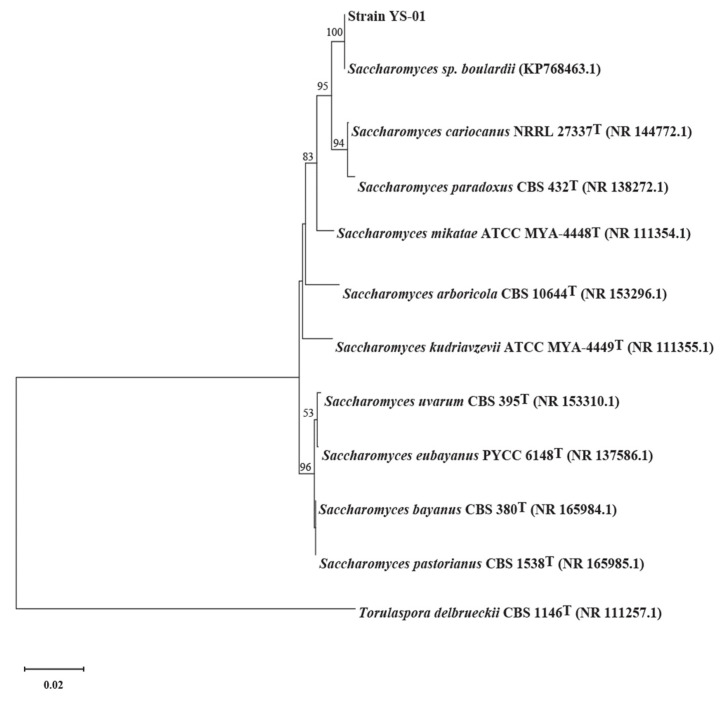
Phylogentic tree of strain-YS01 based on ITS sequences by the neighbor-joining approach.

The composition and function of genes are important for information on microbial physiological and biochemical behavior, which is significant for understanding the biology behind the fermentation reaction and targeting modification to manufacture superior fermentation products [29]. To this end, we de novo sequenced, assembled, and annotated a draft genome of *S. boulardii*-YS01. The phylogenetic tree based on the ITS rDNA sequence data is illustrated in Figure 3.

The total number of bases after quality control was 1,083,269,987 bp. We evaluated the quality of the preliminary assembled genome sequences, and the results showed good quality assembly for further downstream analysis, which can be seen in Figure S5. Plus, the assembly process produced 153 scaffolds, where the total length of the scaffold was 11,541,211 bp. The final genome of YS01 was assembled into 374 contigs after genome assembly, correction, and optimization. The total length of all the assembled contigs was 11,538,623 bp with a GC content of 38.05%, encoding 4275 genes with an N50 value of 100,937 bp. The total length of the predicted protein-coding genes (CDS) was 9,367,056 bp with an average size of 2191.12 bp, giving a coding intensity of 81.16%. Analysis revealed 282 tRNA genes and 2 rRNA operons in the genome. All the detailed parameters are shown in Table 3. Of the 4275 protein-coding sequences, 1369 could be assigned to 23 different types of COG, which lucidly reflect the organism’s efficient energy production and conversion, amino acid transport and metabolism, secondary metabolites biosynthesis, transport and catabolism and inorganic ion transport and metabolism, etc. (Figure S6A). In addition, 17,234 CDS of YS01 were annotated in a Non-Redundant Protein Sequence Database, 16,244 CDS in Swiss-prot, 2961 CDS in Pfam, and 2441CDS in the GO. Table S1 shows the annotation statistics of gene-encoded proteins in different databases.

The result of the KEGG pathway and secondary classification statistical histogram is shown in Figure S6B. A total of 2388 genes were annotated in KEGG, which was involved in six categories, in which 79 genes participated in the pathways of the metabolism of cofactors and vitamins. In short, the genome scan map supplied a reference for discussing genetic determinants behind the biotechnological potential of *S. boulardii* strain-YS01. 

### 3.4. Targeted Metabolic Analysis Combined with Gene Anlysis for Proposing the Formation Pathway of NMN in S. boulardii-YS01

On account of the ambiguous interaction among five analytes in *S. boulardii*-YS01, we further carried out pathway analysis in MetaboAnalyst5.0 with quantitative data and identified the metabolic pathway in the KEGG database (Figure S7B). Genome sequencing and functional annotation were employed to search for the related functional genes and formation pathways of five analytes in YS01. The gene ID and functional annotation of major target-acting enzymes are exhibited in Table 4.

**Figure 4:**
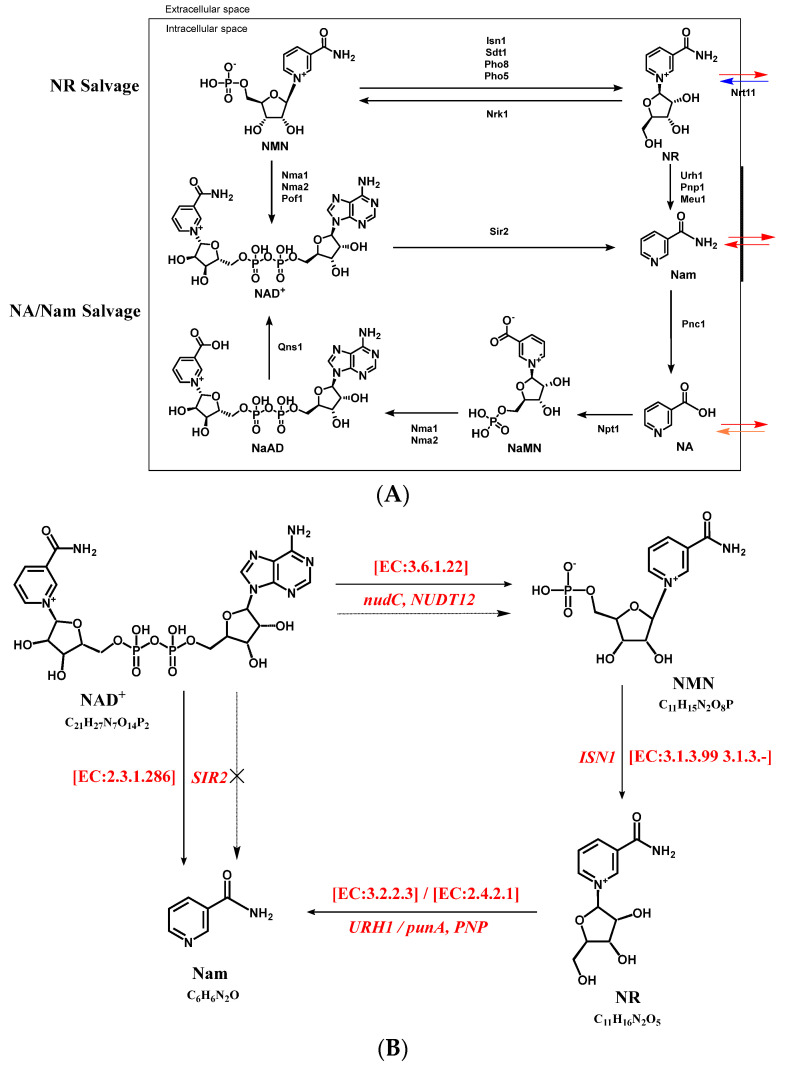
The pivotal pathways of the production of *β*-nicotinamide mononucleotide (NMN), (**A**) The reported pathways involving the production of NMN in *S. cerevisiae*. In the nicotinamide riboside (NR) salvage pathway, NR is converted to NMN by nicotinamide riboside kinase 1 (Nrk1), which helps to synthesize nicotinamide adenine dinucleotide (NAD^+^) using Nma1, Nma2, and Pof1. The nicotinic acid (NA)/nicotinamide (Nam) salvage pathway produces nicotinic acid mononucleotide (NaMN) upon conversion of NA, which is then metabolized to diamino-NAD^+^ (NaAD) and NAD^+^ using Nma1, Nma2, and Qns1, respectively. Urh1/Pnp1-mediated routes responsible for the utilization of exogenous NR generated from NMN by the nucleotidase activities of Isn1 and Sdt1 in the cytosol. The orange arrow illustrates exogenous uptake of NA from yeast growth media. The blue arrow shows transporters of NR by Nrt11. The red arrows represent the mechanisms of these pathways that remain largely unclear. Outside the box shows the extracellular space, while the inside box stands for the intracellular space. (**B**) The proposed formation pathway of NMN in *S. boulardii*-YS01. The dotted arrows refer to Nam-mediated NMN generation pathways in YS01. The number on the arrow is the enzyme number, and the name of the corresponding gene is marked below or inside the arrow. Notes: Nma1/2, NaMN/NMN adenylyltransferase. Qns1, glutamine-dependent NAD^+^ synthetase. Npt1, nicotinic acid phosphoribosyl transferase. Pnc1, nicotinamide deamidase. Sir2 family, NAD^+^-dependent protein deacetylases. Urh1, Pnp1, and Meu1, nucleosidases. Nrk1, NR kinase. Isn1 and Sdt1, nucelotidases. Pho8 and Pho5, phosphatases. Pof1, NMN adenylyltransferase. Nrt11, NR transporter.

Prior works have already certified that Nam is deamidated to NA by Pnc1 in *S. cerevisiae* [17,18]. However, the nicotinamidase (Pnc1, EC:3.5.1.19) that directly enables Nam to form NA was not annotated in YS01; even the enzymes that NA interact with Nam, NR, NMN, and NAD+ were not annotated (Figure S7A), leading to difficulties in interpreting and echoing the correlation analysis results. Meanwhile, in the gene annotated pathway of nicotinate and nicotinamide metabolism (Figure S7A), we could see that NA was sidelong generated from quinolinic acid, deamino-NAD^+^ (NaAD), nicotinic acid ribonucleoside or nicotinic acid mononucleotide, which reasonably explained the increase in NA in YS01. Nevertheless, the concrete pathways corresponding to the annotation information still await further verification. As signified in Figure 4A, some scientific studies have reported that NAD^+^ can be synthesized by salvaging NA, Nam, and NR [17,18]. The accepted view of NAD^+^ biosynthesis, where all anabolism flows through nicotinic acid mononucleotide (NaMN), contrasts the fact that we only obtained the annotation in YS01 that NAD^+^ could be generated from NaAD through NAD^+^ synthase (glutamine-hydrolyzing) (QNS1, EC:6.3.5.1) (Figure S7A). Furthermore, in *S. cerevisiae*, the nucleosidases methylthioadenosine phosphorylase (Meu1), uridine hydrolase (Urh1) and purine nucleoside phosphorylase (Pnp) could be utilized by NR to convert itself into Nam [18,37,38]. In *S. boulardii*-YS01, the uridine nucleosidase (URH1, EC:3.2.2.3), and purine-nucleoside phosphorylase (PNP, punA, EC:2.4.2.1) were also annotated where converting NR to Nam triggered elevated intracellular Nam levels. 

Additionally, it is of interest to note that the IMP 5′-nucleotidase (ISN1, EC:3.1.3.99 3.1.3.-) was annotated, which is capable of generating exogenous NR from NMN (Figure S7A), and this property is similar to some yeasts (Figure 4A). In addition, the Nrk1 assimilating NR into NMN was not annotated in YS01, which differs from *S. cerevisiae* (Figure S7A). As for the MNN, the gene coding for Nampt (Nam phosphorybosyltransferase) in higher eukaryotes, including mammals, has been authenticated to convert Nam to NMN [17,35]. Although YS01 does not possess the gene coding for Nampt, the enzyme called NAD^+^ phosphohydrolase (NUDT12, nudC, EC:3.6.1.22), which can convert NAD^+^ to NMN, was annotated in the database. The NAD-dependent histone deacetylase (SIR2, EC:2.3.1.286) was also annotated. Nam has been deemed an inhibitor of Sir2 [39]. According to the signaling pathway annotated in KEGG (Figure S7A), we put forward the addition of exogenous Nam to indirectly promote NMN formation through inhibiting NAD^+^ consumption to a certain extent, which weakened its dependence on Nampt for NMN synthesis. 

Taking the above analysis results into consideration, we ultimately obtained a chain of metabolism pathway involving NMN, NR, NA, Nam and NAD^+^ and the biosynthetic path of NMN via Nam in YS01 was suggested (Figure 4B), which could provide a molecular cognition and modification target to further construct the genetically engineered strain to vastly increase the generation of NMN. 

### 3.5. Confirmation of Appropriate Nam Addition Concentration for NMN Generation

In light of Nam’s role in promoting NMN production, the established analytical method was applied to detect NMN content and ascertain the optimal amount of Nam addition to generate NMN in *S. boulardii*-YS01. 

**Figure 5:**
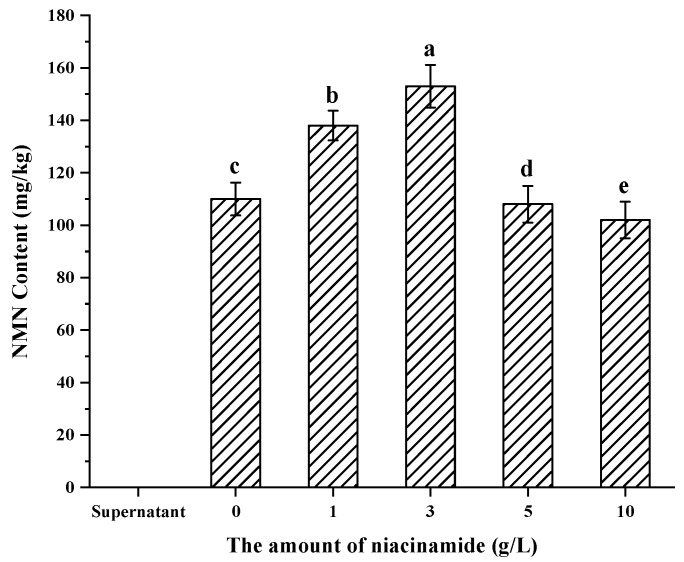
Content of NMN at different nicotinamide addition concentrations in *S. boulardii*-YS01 (*n* = 6). Data in the figure are the mean ± SD. Different lowercase letters mean significant difference (*p* < 0.05).

What makes sense is that we found 3 g/L of Nam evidently heightened the content of NMN in YS01 when comparing low, medium, and high addition amounts, while the excessive addition of Nam was prejudicial to the production of NMN (Figure 5). The optimization of concentration confirmed that Nam addition enabled the stimulation of NMN yield. The optimal Nam addition concentration was 3 g/L, which increased by 39% from the initial level of NMN in YS01. To sum up, the enhancement of NMN in YS01 could be achieved by metabolic supplementation using Nam in the culture medium, which is of certain guiding significance for the industrial mass production of NMN. 

## 4. Conclusions

The natural occurrence of NMN in different foods and living organisms, along with its potential benefits for human health, has attracted much concern. Investigating the content of NMN in latent dietary supplements and increasing its production undoubtedly justifies the work effort. In this research, we reported on the simultaneous quantitation of NMN, NR, NA, Nam, and NAD^+^ from different growth periods in *S. boulardii*-YS01 by means of the UPLC-ESI-QqQ-MS/MS method. The average content of NMN was 5~100 fold higher in YS01 compared to some ordinary foods, which was up to 103.40 mg/kg, and testifies that YS01 could be an alternative source of NMN production. The metabolism and biodistribution of NMN, NR, NA, Nam, and NAD^+^ in various tissues and within cells remain poorly understood. The draft genome sequencing manifested the biotechnological potential of YS01. Through the targeted analysis of the five analytes and combined with enzyme gene function annotation, the metabolism pathway of NMN involving NR, Nam, and NAD^+^ was proposed, which provides basic cognition of Nam-mediated NMN formation in YS01. The results of concentration optimization further show that the appropriate exogenous addition of Nam availably increased the production of NMN in YS01 by 39% on average, and the optimal amount of Nam was 3 g/L. These findings furnish preliminary guidance for the genetic engineering modification of biosynthetic NMN in YS01 and contribute to the exploitation of NMN as a potential food ingredient to improve diets.

## Figures and Tables

**Table 1:** The MRM parameters of five standard compounds.

Compound	Precursor Ion [M + H]^+^ (*m*/*z*)	Product Ions (*m*/*z*)	Fragmentor Voltage (V)	Collision Energy (eV)
NMN	335.1	123.1 */97.2	380	12/22
NR	255.1	123.2 */80.1	380	10/45
NA	124.0	80.1 */78.1	380	25/25
Nam	123.1	80.1 */53.3	380	23/42
NAD^+^	664.1	136 */427.9	380	50/30

Notes: “*” means the quantitative ion. Abbreviations: multiple reaction monitoring (MRM), *β*-nicotinamide mononucleotide (NMN), nicotinamide riboside (NR), nicotinic acid (NA), nicotinamide (Nam), nicotinamide adenine dinucleotide (NAD^+^).

**Table 2:** Method validation using *S. boulardii*-YS01 in stationary phase.

Analytes	Linear Equation	R^2^	Recoveries,% (RSD,%) *n* = 6 Spiked Level (mg/kg)			LOQ (μg/kg)	LOD (μg/kg)
			10	50	100		
NMN	Y = 287.42X + 1536.15	0.9994	76% (4.6)	78% (6.2)	89% (7.1)	0.055	0.016
NR	Y = 21,162.59X + 3489.69	0.9999	70% (3.4)	82% (5.7)	75% (4.9)	0.003	0.001
NA	Y = 7151.59X + 11687.73	0.9995	72% (3.9)	84% (4.5)	80% (2.7)	0.034	0.010
Nam	Y = 29,987.60X + 12,387.99	0.9996	71% (4.1)	73% (5.6)	82% (3.9)	0.082	0.025
NAD^+^	Y = 90.48X + 199.94	0.9989	85% (5.4)	79% (6.3)	74% (4.8)	0.016	0.005

Abbreviations: *β*-nicotinamide mononucleotide (NMN), nicotinamide riboside (NR), nicotinic acid (NA), nicotinamide (Nam), nicotinamide adenine dinucleotide (NAD^+^).

**Table 3:** General properties and statistics of *S. boulardii*-YS01 genome.

Features	Description
Clean reads bases	1,083,269,987 bp
Scaffolds	153
Total bases in scaffold (Genome Size)	11,541,211 bp
Contigs	374
Total bases in contigs	11,538,623 bp
N50	100,937 bp
GC content	38.05%
Gene coding (CDS)	4275
Gene total length	9,367,056 bp
Gene average length	2191.12 bp
Gene/Genome	81.16%
tRNA	282
rRNA	2
SINEs ^a^	18
LINEs ^b^	348
LTR ^c^	701
Simple repeat	2544
Genes of KEGG	2388
Genes of COG	1369

^a^ SINEs: short interspersed elements. ^b^ LINEs: long interspersed nuclear elements. ^c^ LTR: long terminal repeat.

**Table 4:** The information of gene ID and functional annotation of major target-acting enzymes.

Gene ID	Location	Start	End	Length (bp)	Non-Redundant Protein Sequence Database Description	Gene Name	Swiss-Prot Description	GO ID	COG ID	COG Type	KO ID	Pfam ID
gene0375	Scaffold3	97834	99978	2145	Qns1p	nadE	Glutamine-dependent NAD(+) synthetase OS = Saccharomyces cerevisiae (strain ATCC 204,508/S288c)	GO:0005488; GO:0003824; GO:0110165; GO:0008152; GO:0009987	COG0171	H	K01950	PF00795; PF02540
gene1306	Scaffold10	253886	252160	1727	Npy1p	nudC	NAD-capped RNA hydrolase NPY1 OS = Saccharomyces cerevisiae (strain ATCC 204,508/S288c)	GO:0005488; GO:0003824; GO:0110165; GO:0008152; GO:0009987	COG2816	F	K03426	PF00293
gene1408	Scaffold11	233570	232548	1023	Urh1p	-	Uridine nucleosidase OS = Saccharomyces cerevisiae (strain ATCC 204,508/S288c)	GO:0003824; GO:0008152; GO:0009987	COG1957	F	K01240	PF01156
gene1812	Scaffold16	58366aa	60054	1689	NAD-dependent histone deacetylase SIR2	-	NAD-dependent histone deacetylase SIR2 OS = Saccharomyces cerevisiae (strain ATCC 204,508/S288c)	GO:0005488; GO:0003824; GO:0032991; GO:0110165; GO:0065007; GO:0008152; GO:0009987; GO:0051179; GO:0050896	COG0846	O	K11121	PF02146; PF04574
gene2215	Scaffold21	115232	116167	936	PNP1p Purine nucleoside phosphorylase	punA	Purine nucleoside phosphorylase OS = Saccharomyces cerevisiae (strain ATCC 204,508/S288c)	GO:0003824; GO:0110165; GO:0008152; GO:0009987	COG0005	F	K03783	PF01048
gene3938	Scaffold61	37137	38489	1353	IMP 5′-nucleotidase	-	IMP-specific 5′-nucleotidase 1 OS = Saccharomyces cerevisiae (strain ATCC 204,508/S288c)	GO:0005488; GO:0003824; GO:0008152; GO:0009987	-	-	K18550	PF06437

## Data Availability

Data is contained within the article or supplementary material.

## Supplementary Materials

The following supporting information can be downloaded at: https://www.mdpi.com/article/10.3390/foods12152897/s1, Figure S1: The morphological features of *S. boulardii*-YS01 colonies on agar medium and the Gram-staining micrograph; Figure S2: The chromatograms of five analytes under multiple reaction monitoring mode; Figure S3: The matrix effects of *β*-nicotinamide mononucleotide (NMN), nicotinamide nucleoside (NR), nicotinic acid (NA), nicotinamide (Nam) and nicotinamide adenine dinucleotide (NAD^+^) in *S. boulardii*-YS01 were determined at five dilution factors ranging from 20 to 500; Figure S4: The effect of Nam addition on the growth of *S. boulardii*-YS01; Figure S5: Analysis of GC-depth and K-mer frequency distribution of *S. boulardii*-YS01, (A) GC-depth point diagram, (B) K-mer frequency diagram; Figure S6: Statistical legend of gene annotation classification of *S. boulardii*-YS01, (A) COG function classification, (B) Histogram of KEGG; Figure S7: The pathway analysis of *S. boulardii*-YS01, (A) the signaling pathway based on KEGG annotation (NA generation pathway was circled in red), (B) the pathway analysis based on quantification data; Table S1: The average content of five analytes in different *S. boulardii*-YS01 samples (*n* = 3); Table S2: The annotation statistics of *S. boulardii*-YS01 gene-encoded proteins in different databases.

## Abbreviations

NMN, *β*-nicotinamide mononucleotide; NR, nicotinamide nucleoside; NA, nicotinic acid; Nam, nicotinamide; NAD^+^, nicotinamide adenine dinucleotide; Nrk, nicotinamide riboside kinase; UPLC-ESI-QqQ-MS/MS, ultra-high performance liquid chromatography coupled to triple quadrupole tandem mass spectrometry; MRM, multiple reaction monitoring; LOD, limit of detection; LOQ, limit of quantitation; COG, Clusters of Orthologous Groups; GO, Gene Ontology; KEGG, Kyoto Encyclopedia of Genes and Genomes; NaMN, nicotinic acid mononucleotide; NaAD, deamino-NAD^+^.
