# Supplementary material for: Enhanced Production of β-Nicotinamide Mononucleotide with Exogenous Nicotinamide Addition in Saccharomyces boulardii-YS01
Source: Foods. 2023 Jul 29;12(15):2897. doi: 10.3390/foods12152897 (PMC10418623; doi:10.3390/foods12152897)
Supplement: Supplementary file 1 [file foods-12-02897-s001.zip › foods-2525063-supplementary.pdf]

Supplementary materials for

Enhanced production of *β*-Nicotinamide mononucleotide with exogenous nicotinamide addition in *Saccharomyces boulardii*-YS01

Meijie Song [1], Chunhua Yin [1], Qianqian Xu [1], Yang Liu [1], Haiyang Zhang [1], Xiaolu Liu [1], Hai Yan [1,*]

[1]School of Chemistry and Biological Engineering, University of Science and Technology Beijing, Beijing 100083, China; meijie_song@126.com (M.S.) ; chyin@ustb.edu.cn (C.Y.); qianqianxu@ustb.edu.cn (Q.X.); liuyang@ustb.edu.cn (L.Y.); zhanghy@ustb.edu.cn (H.Z.); xiaoluliu@ustb.edu.cn (X.L.)
*Correspondence: haiyan@ustb.edu.cn (H.Y.)

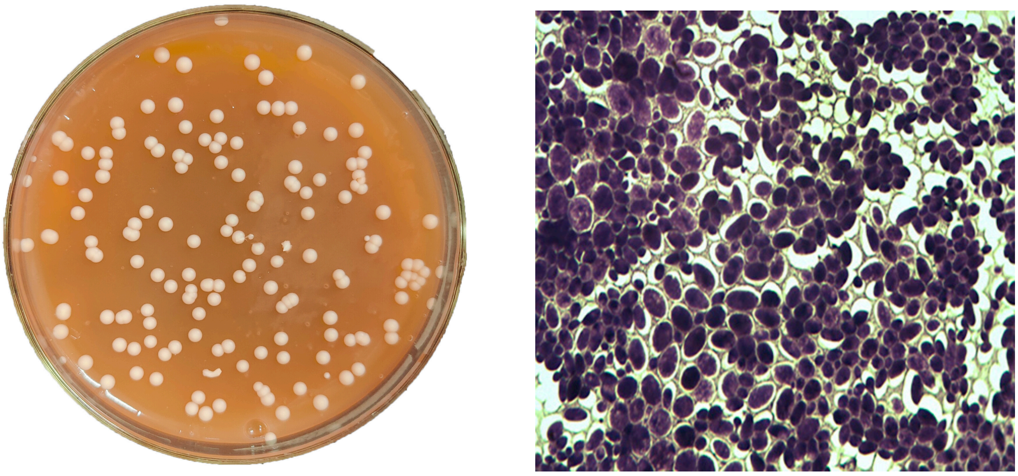

Figure S1. The morphological features of *S. boulardii*-YS01 colonies on agar medium and Gram-staining micrograph.

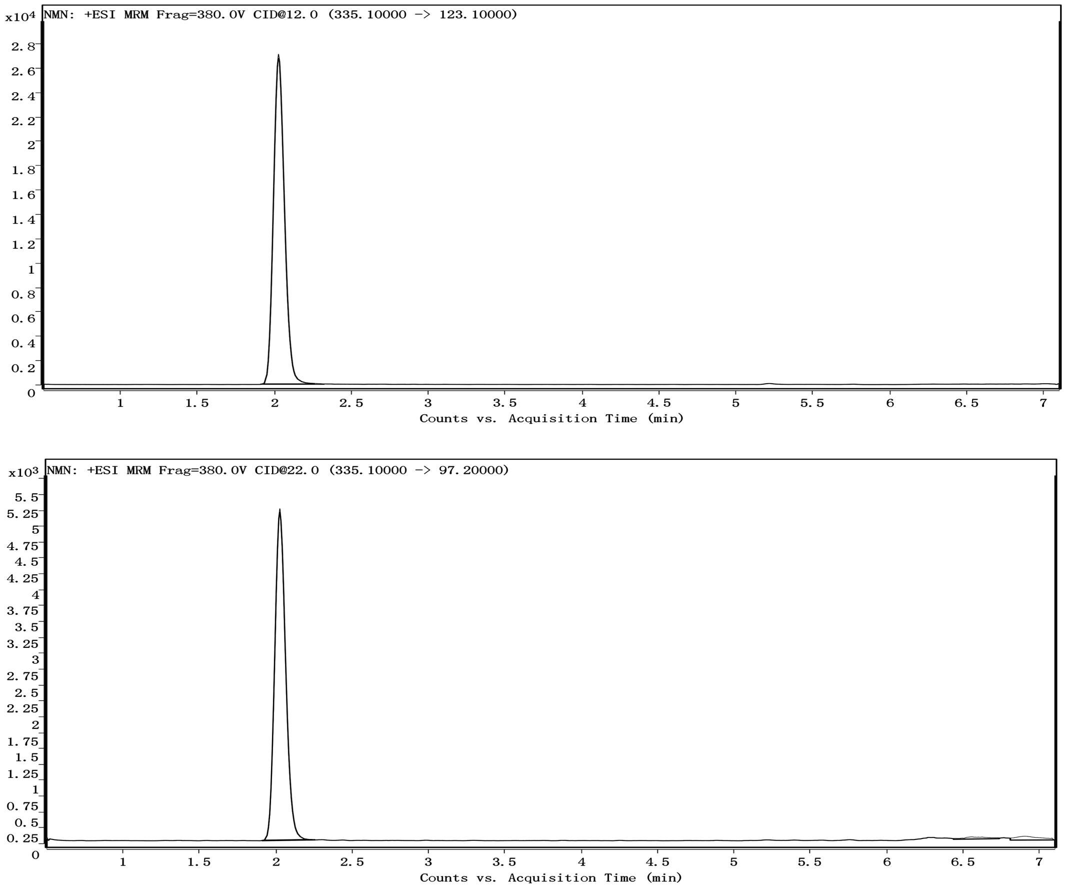

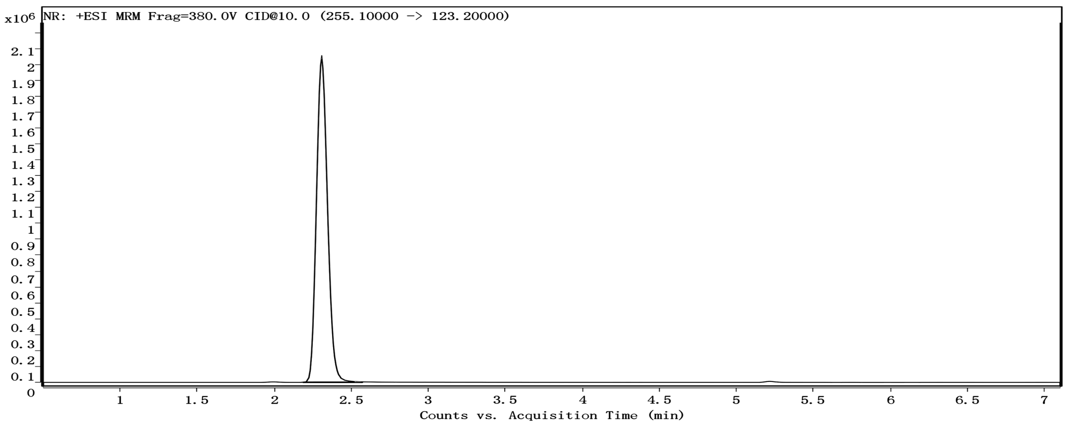
x10^6
NR: +ESI MRM Frag=380.0V CID@10.0 (255.10000 -> 123.20000)
Counts vs. Acquisition Time (min)

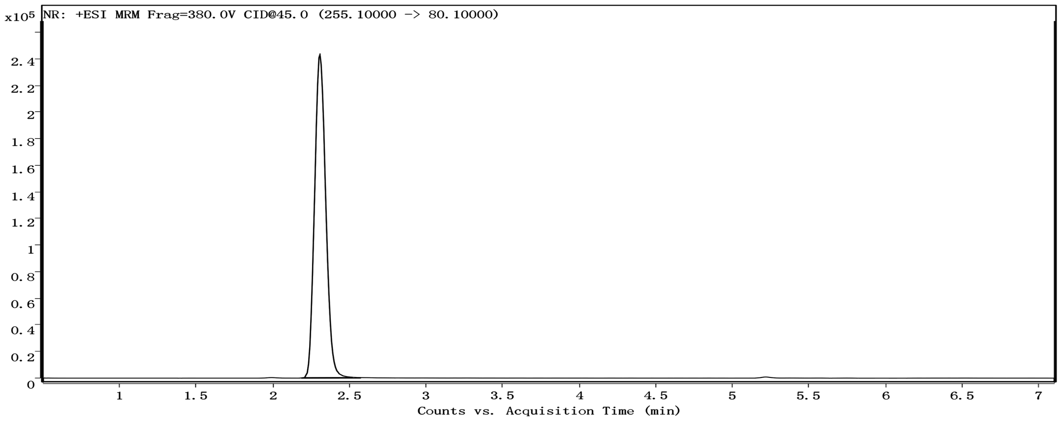
x10^5
NR: +ESI MRM Frag=380.0V CID@45.0 (255.10000 -> 80.10000)
Counts vs. Acquisition Time (min)

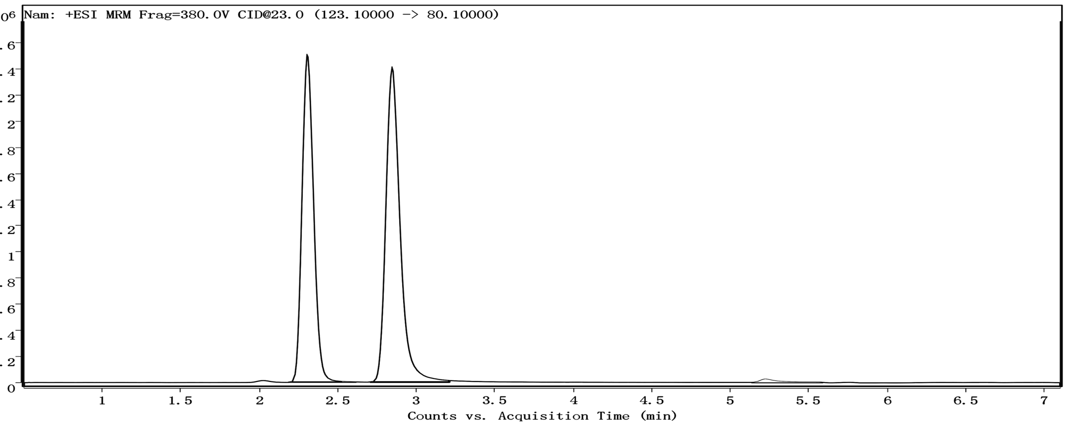
x10^6
Nam: +ESI MRM Frag=380.0V CID@23.0 (123.10000 -> 80.10000)
Counts vs. Acquisition Time (min)

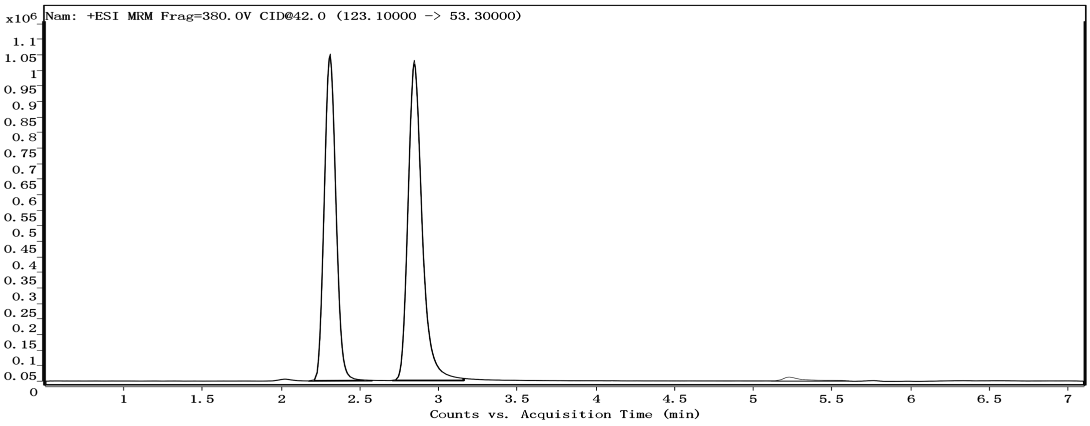
x10^6
Nam: +ESI MRM Frag=380.0V CID@42.0 (123.10000 -> 53.30000)
Counts vs. Acquisition Time (min)

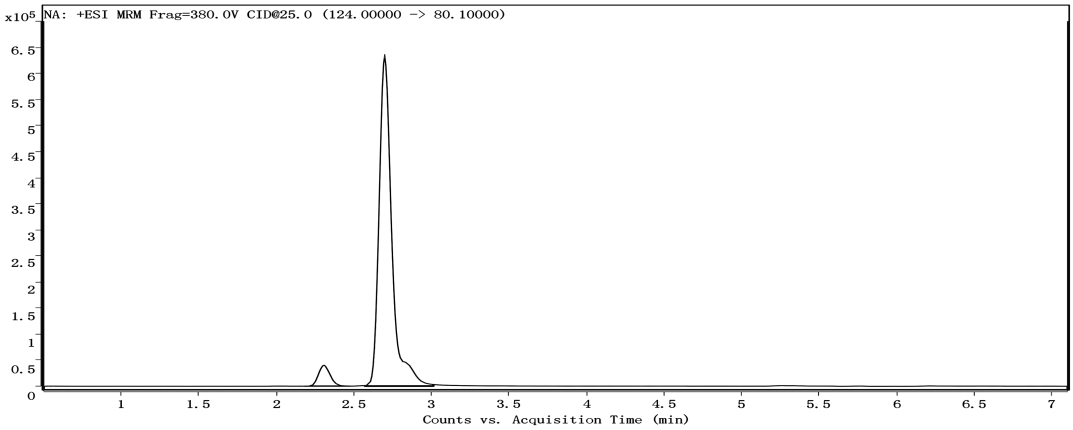
x10^5 NA: +ESI MRM Frag=380.0V CID@25.0 (124.00000 -> 80.10000)
Counts vs. Acquisition Time (min)

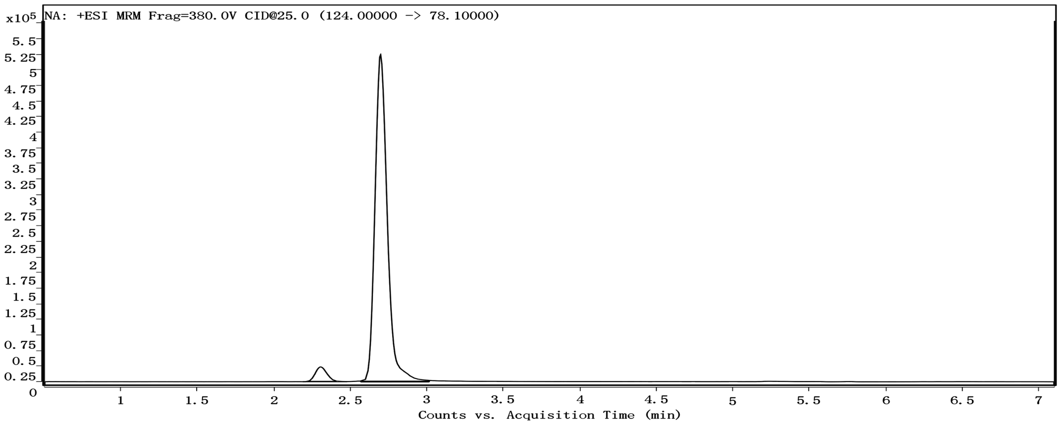
x10^5 NA: +ESI MRM Frag=380.0V CID@25.0 (124.00000 -> 78.10000)
Counts vs. Acquisition Time (min)

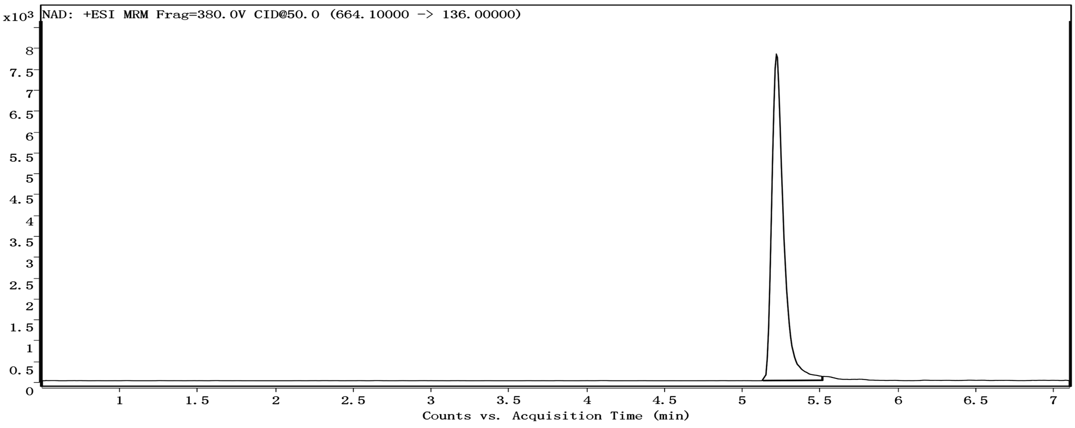
x10^3 NAD: +ESI MRM Frag=380.0V CID@50.0 (664.10000 -> 136.00000)
Counts vs. Acquisition Time (min)

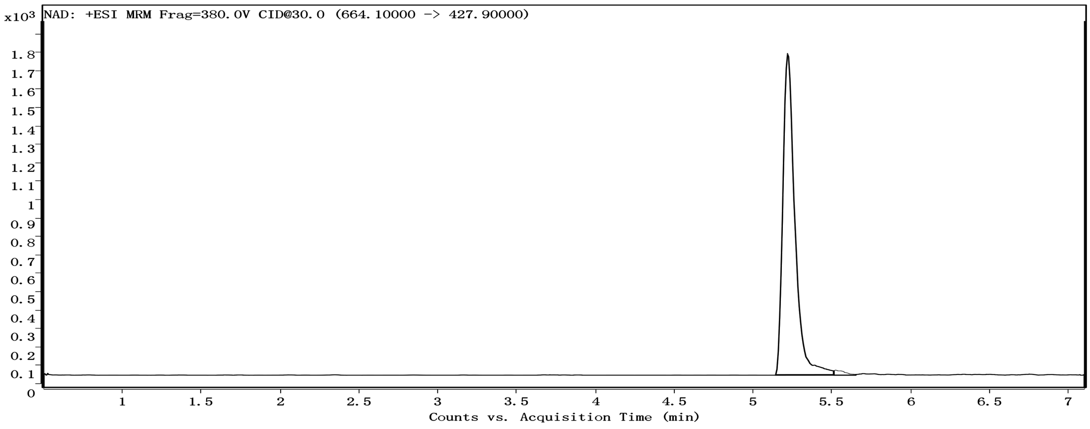
x10^3 NAD: +ESI MRM Frag=380.0V CID@30.0 (664.10000 -> 427.90000)
Counts vs. Acquisition Time (min)

Figure S2. The chromatograms of five analytes under multiple reaction monitoring mode.

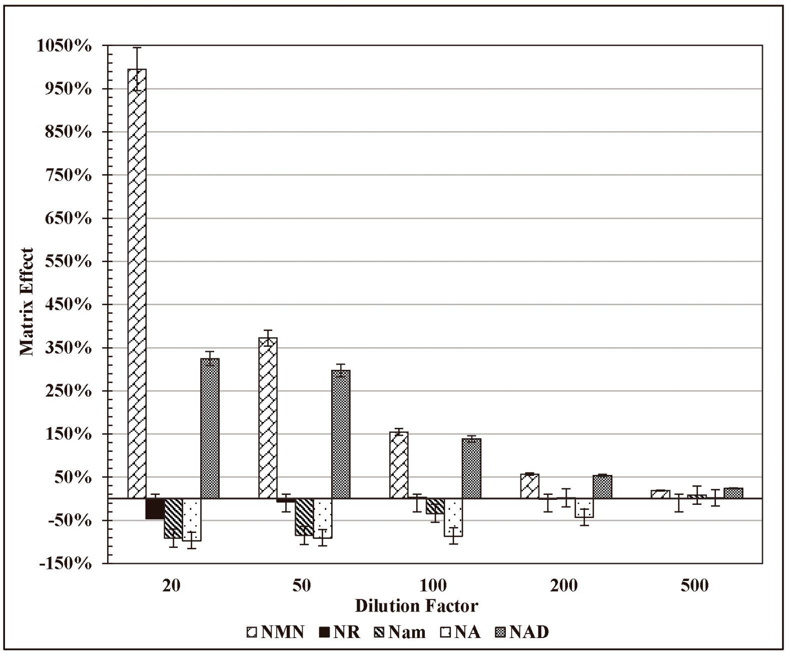

Figure S3. The matrix effects of β-nicotinamide mononucleotide (NMN), nicotinamide nucleoside (NR), nicotinic acid (NA), nicotinamide (Nam) and nicotinamide adenine dinucleotide (NAD+) in *S. boulardii*-YS01 were determined at five dilution factors ranging from 20 to 500.

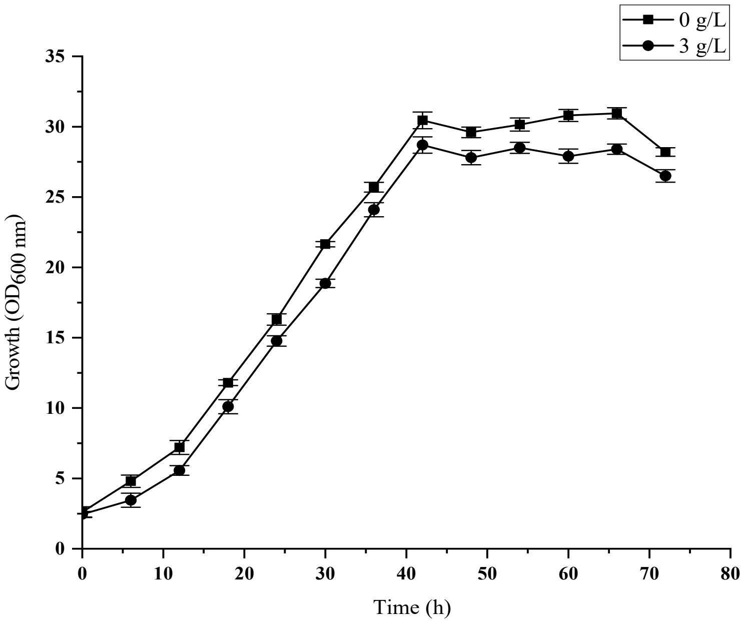

Figure S4. The effect of Nam addition on the growth of *S. boulardii*-YS01.

(**A**)

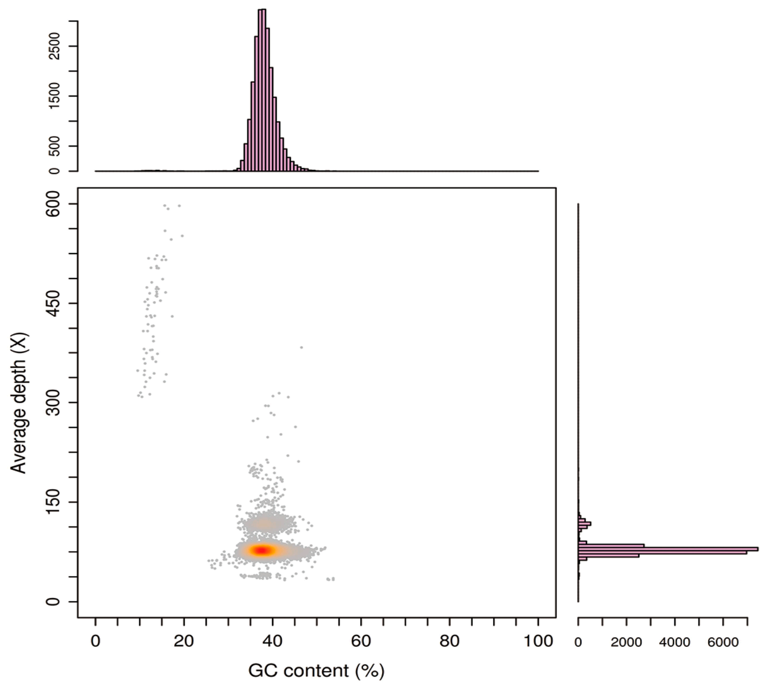

(**B**)

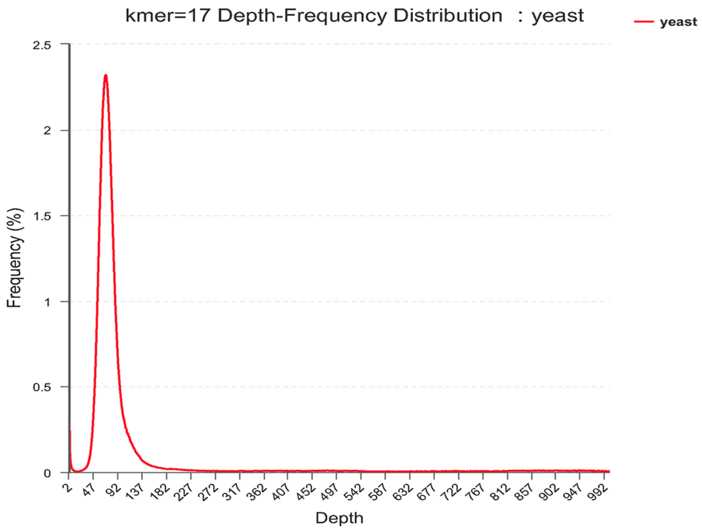

Figure S5. Analysis of GC-depth and K-mer frequency distribution of *S. boulardii*-YS01, (**A**) GC-depth point diagram, (**B**) K-mer frequency diagram.

(**A**)

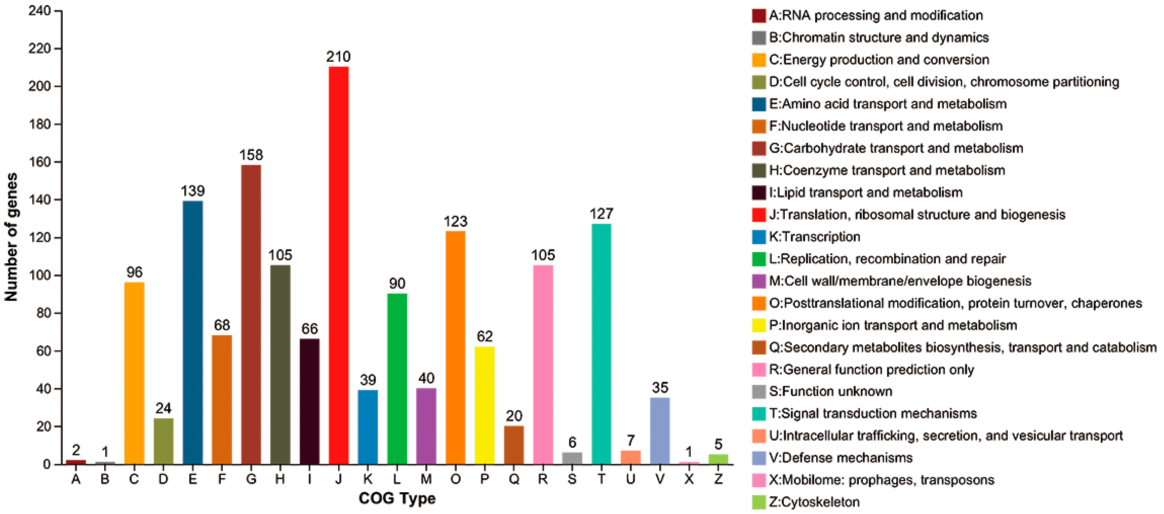

(**B**)

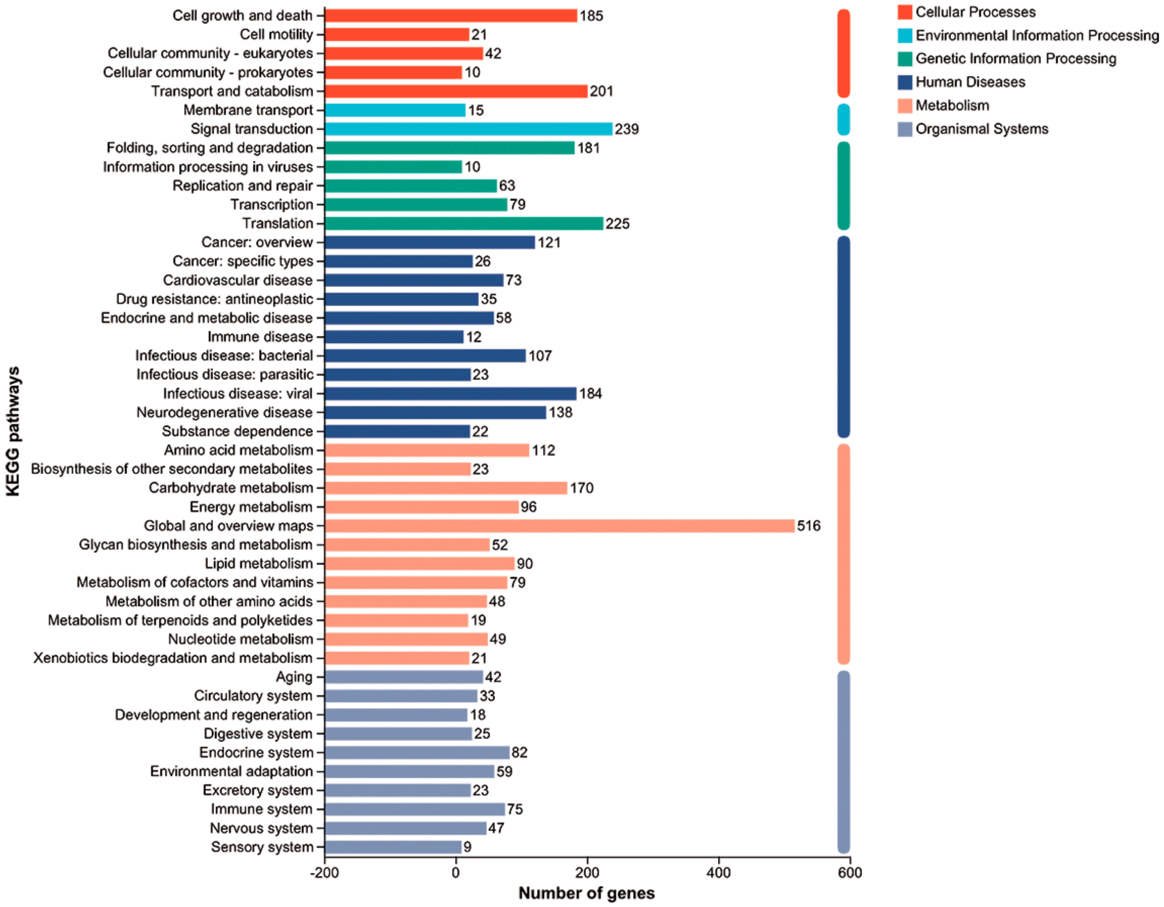

Figure S6. Statistical legend of gene annotation classification of *S. boulardii*-YS01, (**A**) COG function classification, (**B**) Histogram of KEGG.

(**A**)

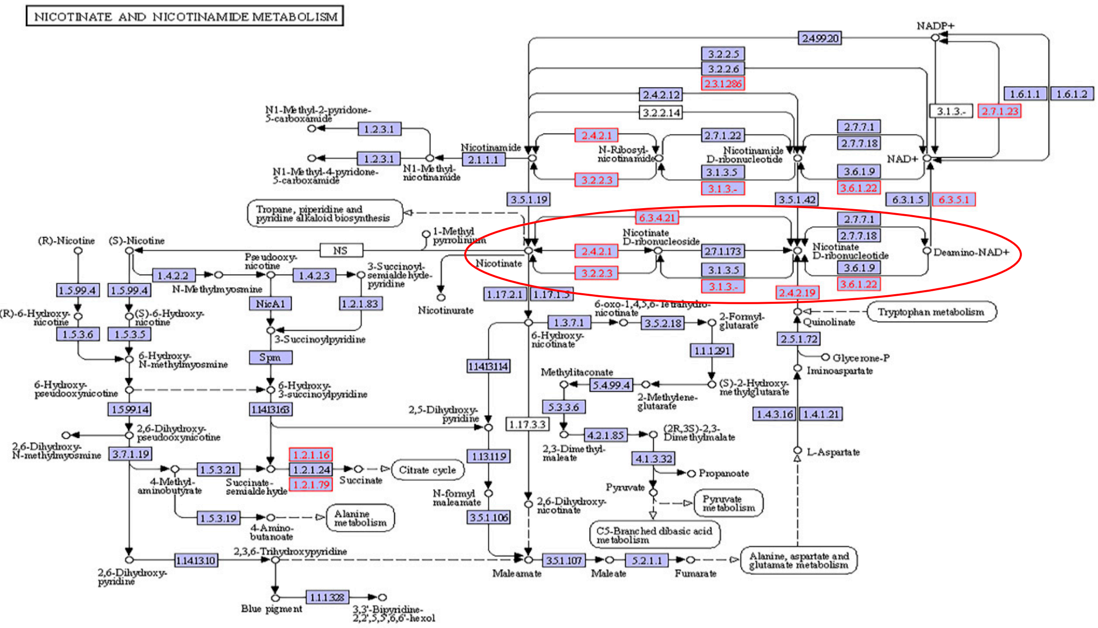

(**B**)

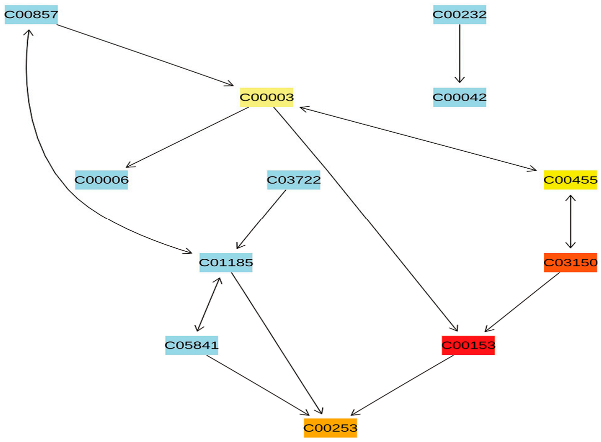

Query	Hit	HMDB	PubChem	KEGG	Details
β-Nicotinamide mononucleotide	Nicotinamide ribotide	HMDB0000229	14180	C00455	
nicotinamide riboside	Nicotinamide riboside	HMDB0000855	439924	C03150	
nicotinic acid	Nicotinic acid	HMDB0001488	938	C00253	
nicotinamide	Niacinamide	HMDB0001406	936	C00153	
nicotinamide adenine dinucleotide	NAD	HMDB0000902	5893	C00003	

Figure S7. The pathway analysis of *S. boulardii*-YS01, (**A**) the signaling pathway based on KEGG annotation (NA generation pathway was circled in red), (**B**) the pathway analysis based on quantification data.

Table S1 The average content of five analytes in different *S. boulardii*-YS01 samples (n=3).

Sample	Group	NMN	NR	NA	Nam	NAD^+
0-1 h	0	0.24 ± 1.60	0.52 ± 1.21	0.3589 ± 4.48	0.41 ± 2.21	4.81 ± 5.61
0-12 h	0	32.12 ± 4.32	8.54 ± 1.27	4.89 ± 9.56	6.59 ± 1.42	46.50 ± 6.26
0-24 h	0	57.93 ± 5.66	17.10 ± 2.63	19.26 ± 13.98	12.91 ± 2.01	29.49 ± 4.32
0-36 h	0	71.85 ± 6.43	15.98 ± 2.90	14.46 ± 10.90	12.39 ± 1.98	37.88 ± 4.10
0-48 h	0	100.57 ± 8.72	14.13 ± 3.36	0.83 ± 6.97	10.44 ± 1.69	56.02 ± 5.13
0-60 h	0	98.78 ± 6.12	17.59 ± 2.10	0.97 ± 8.56	13.59 ± 4.12	86.97 ± 6.02
0-72 h	0	103.40 ± 5.90	14.40 ± 2.93	7.63 ± 11.75	11.30 ± 1.72	92.37 ± 4.73
3-1 h	3	0.26 ± 2.70	0.62 ± 2.09	0.86 ± 5.76	0.28 ± 1.40	3.85 ± 3.41
3-12 h	3	33.84 ± 5.34	8.30 ± 1.54	104.41 ± 8.41	6.28 ± 2.08	70.76 ± 5.46
3-24 h	3	58.13 ± 3.20	20.38 ± 2.36	220.51 ± 9.10	15.14 ± 2.70	42.58 ± 6.04
3-36 h	3	86.79 ± 5.31	25.14 ± 3.46	193.30 ± 12.87	19.45 ± 2.35	43.06 ± 4.31
3-48 h	3	126.71 ± 6.92	16.91 ± 2.30	145.47 ± 13.60	13.55 ± 2.20	57.33 ± 3.24
3-60 h	3	137.36 ± 8.40	15.82 ± 1.49	167.37 ± 9.89	11.82 ± 3.49	62.52 ± 5.39
3-72 h	3	128.75 ± 6.75	15.56 ± 1.75	189.54 ± 10.70	11.52 ± 3.10	104.4 ± 6.86

Notes: Values are mean ± standard deviation of at three replicates. 0 means without Nam addition and 3 means 3 g/L of Nam addition in the culture medium of *S. boulardii*-YS01. Abbreviations: *β*-nicotinamide mononucleotide (NMN), nicotinamide riboside (NR), nicotinic acid (NA), nicotinamide (Nam), nicotinamide adenine dinucleotide (NAD^+).

Table S2 The annotation statistics of *S. boulardii*-YS01 gene-encoded proteins in different databases.

**Database name**	Non-Redundant Protein Sequence Database	Swiss-Prot	Pfam	Gene Ontology
**Gene number**	17234	16244	2961	2441
